# A Case of Intestinal Obstruction in Pregnancy Diagnosed by MRI and Treated by Intravenous Hyperalimentation

**DOI:** 10.1155/2016/8704035

**Published:** 2016-11-24

**Authors:** Atsushi Daimon, Yoshito Terai, Yoko Nagayasu, Atsuko Okamoto, Takumi Sano, Yusuke Suzuki, Kazuyoshi Kanki, Daisuke Fujita, Masahide Ohmichi

**Affiliations:** Department of Obstetrics and Gynecology, Osaka Medical College, Osaka, Japan

## Abstract

Intestinal obstruction in pregnancy is rare and is mainly caused by prior pelvic surgery. We herein report a case of intestinal obstruction in a pregnant female with a history of laparoscopic myomectomy, who presented with hypogastric pain, abdominal distension, and vomiting at 26 weeks of gestation. A simple intestinal obstruction was diagnosed by MRI. Conservative treatments, including intravenous hyperalimentation and the placement of an ileus tube, were provided and her abdominal symptoms improved for 14 days. After restarting oral intake, she had no abdominal symptoms. She gave birth to a 2,146 g female infant by caesarean section at 37 weeks and 1 day of gestation. Although an area of cicatrization, which was thought to have been the starting point of the occlusion that caused the intestinal obstruction, was found, the excision of the small intestine was not necessary. Her postoperative course was uneventful. Intestinal obstruction requires a prompt diagnosis and aggressive intervention may be necessary to minimize the morbidity and mortality associated with this rare complication of pregnancy. MRI can be safely used during pregnancy to diagnose intestinal obstruction and intravenous hyperalimentation may improve the maternal and fetal prognoses.

## 1. Introduction

Intestinal obstruction in pregnancy is rare, and it is mainly caused by pelvic surgery prior to conception. The appropriate diagnosis and management of intestinal obstruction during pregnancy are of paramount importance, as it is associated with significant maternal and fetal mortality [1]. The diagnosis and treatment of intestinal obstruction in pregnant patients are similar to those in the nonpregnant patients [2]. Pregnant women with an inadequate oral intake or underlying disease states that require complete bowel rest should be treated with parenteral nutrition by intravenous hyperalimentation [3].

We herein report a case of intestinal obstruction in pregnancy in a patient who was diagnosed with a simple intestinal obstruction by magnetic resonance imaging (MRI) and who was safely delivered at term following successful treatment using conservative therapy, which included intravenous hyperalimentation and an ileus tube.

## 2. Case Report

**Figure 1:**
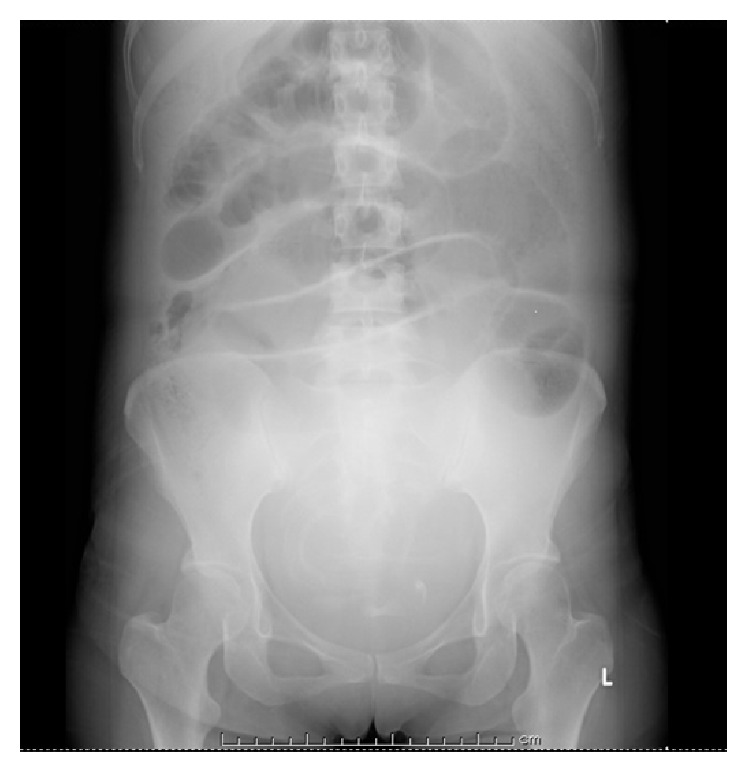
Abdominal X-ray showed air-fluid level of small intestine and dilated intestine.

**Figure 2:**
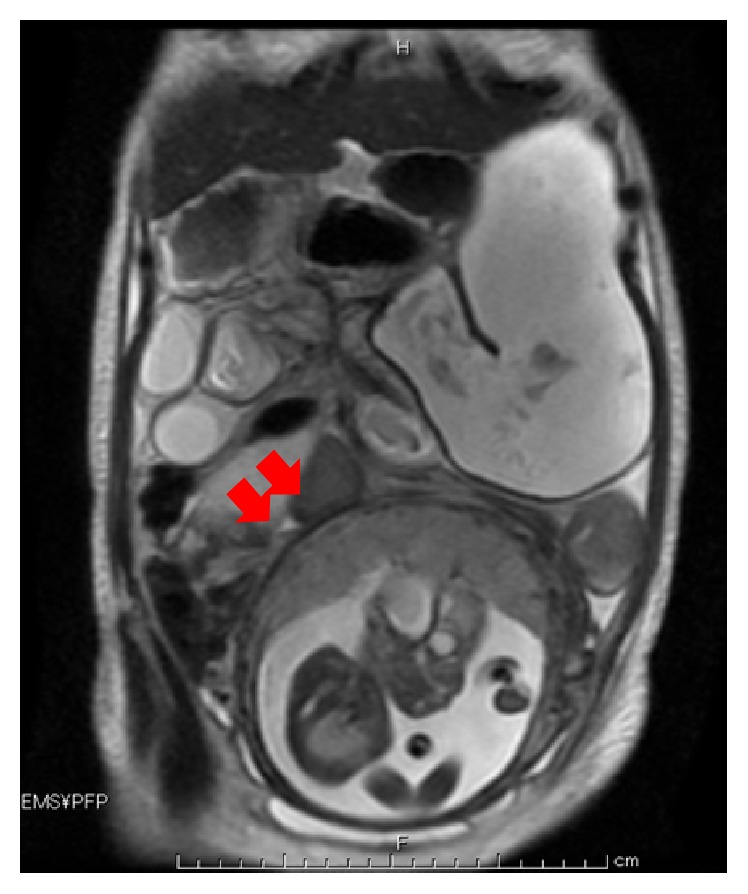
MRI showing the narrowing of the distal extent of the ileum and the dilated proximal intestine (arrows).

A 37-year-old woman (Gravida 0, Para 0) was referred to our institution at 25 weeks and 4 days of gestational age with symptoms of abdominal pain and vomiting. She had a history of appendectomy and laparoscopic myomectomy. Her body temperature was 36.6°C, her heart rate was 65 beats/min, and blood pressure was 120/67 mmHg. Her abdomen was soft, flat, and without tenderness; her bowl sounds were enhanced. She had a WBC count of 12,380/*μ*L and CRP level of 0.58 mg/dL. The other laboratory data were within the normal limits. Her cervix was uneffaced and the cervical os was closed. Because her symptoms were improved by intravenous feeding and the administration of an antispasmodic agent, she was diagnosed by acute gastroenteritis and discharged to return home. At that time, she had no sign of threatened labor. However, her abdominal pain and vomiting did not improve and she was hospitalized the next day. On the third day of hospitalization, her symptoms worsened. Her abdomen became distended and bowel sounds were absent on physical examination. Abdominal X-rays showed air-fluid level of small intestine (Figure 1), and she was diagnosed with small bowel obstruction. It was thought that her obstruction had been caused by adhesion. Cardiotocography revealed irregular uterine contraction. She was treated with an intravenous infusion, antibiotics, antiemetics, and a *β*-2 stimulant for tocolysis. On the fourth day of hospitalization, she exhibited frequent bilious vomiting. MRI confirmed the dilation of loops of the small bowel, with the starting point of the obstruction located in the ileac part, contacting the right side of the uterus (Figure 2), but no sign of strangulation obstruction was observed.

**Figure 3:**
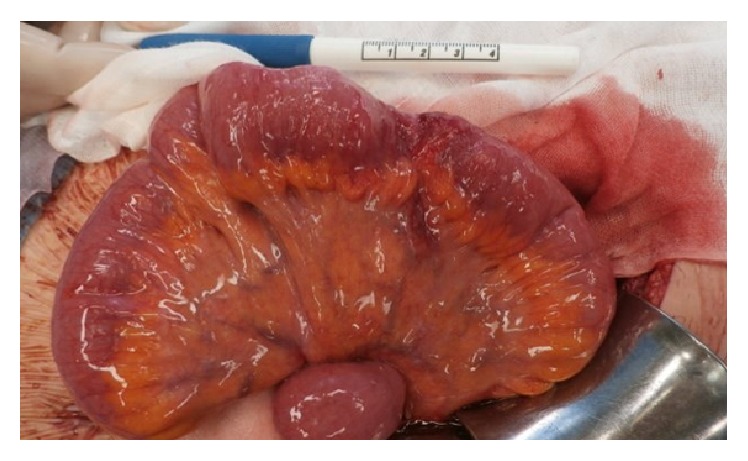
Redness and cicatrization of the small intestine and the mesenterium were observed 20 cm cephalad from the terminal ileum, which was thought to be the starting point of obstruction.

Conservative treatment was continued, a nasal ileus tube and central venous catheter were inserted and total parenteral nutrition (TPN) was initiated. TPN was used from the 8th day to the 20th day of hospitalization and was not changed to PPN. Her abdominal symptoms gradually improved, and on the 8th day of hospitalization (gestational age: 26 weeks and 6 days) she showed flatus. On the 14th day of hospitalization (gestational age: 27 weeks and 4 days), she resumed oral intake with fluid food. After the initiation of oral intake, her abdominal symptoms stabilized. The ileus tube and central venous catheter were removed on the 17th and 20th days of hospitalization, respectively. She felt irregular uterine contractions, and her cervical length shortened to 15 mm. Therefore, she remained hospitalized for tocolysis between the 21st and 80th days. The remaining course of her pregnancy was uneventful, without a relapse of the intestinal obstruction relapse or any signs of threatened labor. As she did not request vaginal delivery after myomectomy, she received caesarean section on the 81st day of hospitalization (gestational age: 37 weeks and 1 day) and delivered a healthy baby girl weighing 2,146 g. The baby had a good APGAR-score 9 (1 min)/10 (5 min) and no fetal distress (umbilical artery gas, pH 7.301; BE, 0.1 mmol/L). The mesenterium and the uterus fundus were covered with fibrous material.  Figure 3 shows redness and cicatrization of the small intestine and the mesenterium, 20 cm cephalad from the terminal ileum, which was thought to be the starting point of the obstruction. We did not perform resection at that time because we confirmed that the small intestine was not constricted and that the distal ileum had not collapsed. We noted no abdominal-pelvic adhesion during caesarean section. The baby was admitted to the neonatal intensive care unit due to a low birth weight. The mother and her baby had no major medical problems after operation and were discharged from our hospital at 7 days after the operation. In the two years of follow-up, there has been no sign of recurrence of the intestinal obstruction.

## 3. Discussion

We reported the case of a primiparous woman with simple intestinal obstruction that was diagnosed by MRI, who was successfully treated using a conservative approach that included TPN with intravenous hyperalimentation. She was able to safely deliver an infant at term.

The diagnosis and management of acute abdominal pain during pregnancy are often challenging. The most common nonobstetric abdominal conditions that require surgical in pregnancy are acute appendicitis, bowel obstruction, cholecystitis, and pancreatitis. Intestinal obstruction in pregnancy is rare and is the second most common nonobstetric reason for surgical intervention during pregnancy [1]. The incidence of intestinal obstruction in pregnancy ranges from 1 in 1,500 to 1 in 66,000 deliveries [2, 4]. Adhesion from previous abdominal surgery, which is implicated in 60–70% of cases of mechanical obstruction, is most common etiology of this condition. An additional 25% of cases result from volvulus, while intussusception causes 5% of cases [5, 6]. The rates at which intestinal obstruction due to adhesion are detected during the first, second, and third trimester of pregnancy and postpartum are 6%, 27%, 44%, and 21%, respectively [7]. The common symptoms of intestinal obstruction in pregnancy include abdominal pain (98%), vomiting (82%), and constipation (30%). Abdominal tenderness and abdominal peristalsis are observed in 71% and 55% of the patients, respectively [1]. The further displacement of the abdominal organs as pregnancy progresses results in the presentation of symptoms at atypical locations [5].

Although rare, intestinal obstruction during pregnancy is associated with a maternal mortality rate 6–20%, while fetal loss occurs in 26–50% of such cases [4]. The early diagnosis and successful treatment of intestinal obstruction during pregnancy are therefore paramount for maintaining the wellbeing of both the mother and fetus [6]. The diagnosis of intestinal obstruction can be confirmed by a number of modalities. Ultrasound scan may demonstrate fluid-filled bowel loops, and plain abdominal radiography can be helpful for making the diagnosis. Although the reported sensitivity in pregnant patients has been found to be low [5], computed tomography (CT) is the mainstay imaging modality that is applied in the management of small bowel obstruction in nonpregnant patients. In pregnant patients, however, CT exposes the fetus to ionizing radiation. MRI is capable of providing large field-of-view images of maternal abnormalities with excellent soft-tissue contrast and allows for the pancreatic and biliary ducts, blood vessels, and genitourinary tract to be observed without the intravenous administration of a contrast agent. Furthermore, images obtained with MRI do not expose the fetus to ionizing radiation and are often diagnostic without the need for the intravenous administration of contrast material [8]. MRI is useful in the evaluation of pregnant patients with abdominal symptoms, both for delineating the anatomy and for excluding a variety of candidate pathological processes that may give rise to small bowel obstruction [8].

The treatment of intestinal obstruction in pregnant women is similar to that of nonpregnant women. In the absence of signs of peritonitis, conservative treatment should initially be attempted. Since intra-abdominal surgery can lead to premature uterine contractions, tocolytic agents are administered prophylactically when conservative therapy fails and when there are signs of impending bowel strangulation [1]. In the third trimester, if adequate intestinal exposure cannot be obtained, caesarean section must be performed [9]. In the presence of severe malnutrition, pregnancy is associated with an increased risk of spontaneous abortion, congenital malformation, fetal growth restriction, preterm delivery, and perinatal mortality and morbidity [3]. In pregnant women with an inadequate oral intake, underlying disease states that require complete bowel rest or severe illness should be treated with TPN by intravenous hyperalimentation. TPN might promote fetal growth in cases involving fetal growth restriction due to severe maternal nutritional deprivation [10]. TPN can be helpful and lifesaving for malnourished pregnant women [3].

We reported a case of a primiparous woman with a simple intestinal obstruction that was diagnosed by MRI and who was successfully treated using a conservative approach that included TPN with intravenous hyperalimentation. An infant was safely delivered at term. In cases involving pregnant women with a history of abdominal surgery who have symptoms of intestinal obstruction, including abdominal pain, nausea, vomiting, and abdominal distension, the intestinal obstruction should be early diagnosed and treated in the same manner as in nonpregnant women.

## Abbreviation

MRI: Magnetic resonance imaging
WBC: White blood count
CRP: C-reactive protein
TPN: Total parenteral nutrition
CT: Computed tomography.
